# Examining Frailty Phenotype Dimensions in the Oldest Old

**DOI:** 10.3389/fpsyg.2020.00434

**Published:** 2020-03-26

**Authors:** Sara Alves, Laetitia Teixeira, Oscar Ribeiro, Constança Paúl

**Affiliations:** ^1^Abel Salazar Institute of Biomedical Sciences—University of Porto (ICBAS.UP), Porto, Portugal; ^2^Center for Health Technology and Services Research (CINTESIS.ICBAS), Porto, Portugal; ^3^Center for Health Technology and Services Research (CINTESIS.UA), University of Aveiro, Aveiro, Portugal; ^4^Department of Education and Psychology, University of Aveiro, Aveiro, Portugal

**Keywords:** physical frailty, Fried phenotype of frailty, phenotype components, oldest old, frailty dimensions

## Abstract

**Introduction:**

Frailty has been studied among the old population due to its association with negative outcomes. Presently there is no gold standard for measuring frailty, but several studies have used the frailty phenotype of Fried consisting of five components (weakness, slowness, unintentional weight loss, exhaustion, and low physical activity) that classify individuals as robust, pre-frail, or frail, depending on the number of components affected, respectively, zero, one or two, and three or more. This study aims to explore the specific contribution of each of these components to the frailty phenotype in a sample of oldest old community-dwelling individuals.

**Materials and Methods:**

Individuals aged 80+ years old living in the community (*N* = 142) participated in this study. Sociodemographic data (age, sex, educational level, and marital status) and Fried’s frailty phenotype were collected. Descriptive analysis summarized sociodemographic information and the frailty components. Multiple correspondence analysis (MCA) was performed to detect and explore relationships between frailty’s five components.

**Results:**

Participants had a mean age of 88.07 years (*SD* = 5.30 years) and were mainly women (73.9%). The majority of the sample were considered frail (71.8%) and pre-frail (24.7%), and the most recurrent component for both groups was slowness. From the MCA analysis, a two-dimension solution was considered the most adequate, with 53.47% of variance explained. Dimension 1 (32.21% of variance explained) showed weakness as the most discriminant component; dimension 2 (21.26% of variance explained) showed unintentional weight loss as the most discriminant component.

**Discussion:**

Results revealed a high number of pre-frail and frail participants. MCA proved to add an important understanding in examining the frailty phenotype; it revealed weakness as the most discriminant component for dimension 1, suggesting a high association with the frailty phenotype. MCA also identified two main features of frailty: one related with physical features (motor behavioral and grip strength) including weakness, low physical activity, and slowness; and the second related with intrinsic conditions (unintentional weight loss and exhaustion).

**Conclusion:**

This study corroborates the need of a differentiated approach to the frailty phenotype among very old individuals, bringing for consideration the specific influence of its components.

## Introduction

Worldwide trends show an increasing and fast aging population. A longer life expectancy contributes to the increase of individuals aged 80 years and older—the oldest old population. In Portugal, oldest old individuals constitute 5.0% (532,219) of the total population (10,562,178) and 26.5% of the population aged 65+ (2,010,064) (Brandão et al., 2017). Trends show that living longer may lead to a long period of disability and frailty with increasing care demands (Alves et al., 2016).

Frailty has been widely studied among the old population due to its relation with negative outcomes such as falls, institutionalization, hospitalization, and death. Nevertheless, there is not a gold standard to study frailty. Several studies have used Fried’s frailty phenotype (Fried et al., 2001), which defines frailty as the presence of five components: weakness, slowness, exhaustion, low physical activity, and unintentional weight loss. According to this perspective, individuals can be classified as robust, pre-frail, or frail depending on the number of components that they score (0 components, 1–2 components, or ≥3 components, respectively).

Previous research has shown that there is a significant association between increased age and frailty, revealing that the majority of frail individuals are the oldest ones (e.g., Fried et al., 2001; Duarte and Paúl, 2015; Carey et al., 2018; Lewis et al., 2018). Fried’s original study in particular showed that individuals aged 80+ years old represented 34.8% of the frail sample. This number could be higher because there is a large difference between the number of individuals assessed under and above the 80 years old threshold (4,636 versus 681 participants). When analyzing specifically the proportion of frail individuals based on age groups under and above 80 years, Fried’s original study revealed that 18.8% of individuals aged 80 and over were frail in contrast with 5.2% of frail individuals below that age (less than one-third of the frail oldest old participants). Along with this discrepancy, the study did not report information on the proportion of pre-frail individuals in groups under and above 80 years, nor the proportion of components impacted based on pre-frail and frail condition.

Recent studies that used the frailty phenotype revealed that the proportion of frailty among oldest old individuals is particularly high (Duarte and Paúl, 2015; Bieniek et al., 2016) in comparison with younger old individuals (e.g., frailty prevalence increased with age from 31.7% in the 60–69 age group to 67.6% in the 90+ age group, and from 22.5% in the 50–65 age group to 60.4% in the 75+ age group, respectively). These results seem to indicate that the frail condition is very frequent among oldest old individuals and suggests that the frailty phenotype provides low variability within the oldest old subgroup once a large proportion of oldest old individuals are frail. Other studies have already analyzed the components of the frailty phenotype and showed some results in relation to characteristics such as age (Hoogendijk et al., 2015), gender (Bieniek et al., 2016), disability (Papachristou et al., 2017), and mortality (Papachristou et al., 2017). Nevertheless, these studies did not inform about the weight/contribution of each component for the frailty phenotype, and it would be important to understand if all components contribute equally (or not) to the frailty condition and how they interrelate with each other.

In the oldest old group, due to the large proportion of individuals classified as frail (low variability), it would be crucial to determine which components of the frailty phenotype contribute the most to establish the frailty condition. Determining such weights would help to make frailty screening more efficient and more targetable, since the success of interventions, considering frailty as a reversible condition, may depend on the specific components to be addressed. This study aims to explore the structure of the frailty phenotype of Fried and the contribution of each of its components in a sample of oldest old community-dwelling individuals by using multiple correspondence analysis (MCA).

## Materials and Methods

### Design

A non-probabilistic sample was recruited from June of 2017 to August of 2018, in the Metropolitan Area of Porto (North of Portugal). Recruitment was based on the referral of individuals by local NGOs—non-governmental organizations (e.g., day centers and home services) and by using a snowball strategy (Biernacki and Waldorf, 1981), which allowed the identification of cases of interest among people who knew others with similar characteristics and therefore within the scope of the research. A two-stage process was used: first, NGOs were invited to participate in the project. Those that agreed to participate identified possible participants according to a set of inclusion criteria (people aged 80+ years old and living in the community in the Metropolitan Area of Porto). The secretary of each organization then contacted each potential participant in order to ask for authorization for sharing personal data with the research team. After this preliminary consent, the research team contacted the subjects and provided a more detailed description of the study, namely, its objectives and conditions. Those willing to participate were interviewed face-to-face. If the oldest old person had no cognitive ability to respond (e.g., people with dementia), permission to participate was obtained by the legal representative. All participants signed an informed consent form: one for the researcher/interviewer and the other for the participant. The study was approved by the Ethical Committee of the Institute of Biomedical Sciences of Abel Salazar, University of Porto (process no. 188/2017), and authorized by the Portuguese Data Protection Authority (approval no. 1338/2017).

### Measures

– Sociodemographic information: age, sex, education level, and marital status.
– Phenotype of frailty: we assessed five components according the definition of physical frailty proposed by Fried et al. (2001): (i) weakness, (ii) slowness, (iii) unintentional weight loss, (iv) exhaustion, and (v) low physical activity. Regarding the frailty phenotype, participants were considered “frail” if they fulfilled three or more criteria, “pre-frail” if they fulfilled one or two, and “robust” if none of the criteria was fulfilled. The metrics were slightly changed following the procedures used in similar studies with very old individuals (e.g., Gonzalez-Pichardo et al., 2013; Nyunt et al., 2017). In particular:
(i) Weakness was measured using handgrip strength [dynamometer (Takei dynamometer, T.K.K. 5401, Japan)]. Grip strength was tested two consecutive times on both the right and left hands. Analysis used the average peak value across both hands, and the third quartile was considered to classify participants according to their weakness; participants with values < 13.6 kg were considered weak and were categorized as 1, and those who obtained values ≥13.6 kg were categorized as 0, meaning they were not weak (high strength).
(ii) Slowness was evaluated using gait speed by the Timed “Up and Go” test (Podsiadlo and Richardson, 1991). The patient must stand up from an armchair, walk 3 m, turn around, walk back to the chair, and sit down. If the participants took 16.8 or more seconds [Portuguese cutoff for people 80 years and older (Almeida et al., 2017)] to perform the test they were considered to have low mobility and categorized as 1. Participants who were not able to do the walking test were also categorized as 1 (low mobility). Participants who performed the test in less than 16.8 s were categorized as 0, meaning good mobility.
(iii) Unintentional weight loss was evaluated using step 2 of the Malnutrition Universal Screening Tool (Bapen, 2003). Each participant answered about the total unplanned weight loss in the past 3–6 months considering the total of his or her weight. Initially the question was scored as 0 for weight loss < 5%, 1 for weight loss between 5 and 10%, and 2 for weight loss > 10% of the total of weight. Answers were then recoded as 0 for weight loss < 5% and 1 for weight loss ≥5%.
(iv) Exhaustion was assessed using the question “In this last month, do you feel that you have less energy to do the things you want?,” which was categorized as 0 = no exhaustion or 1 = yes exhaustion.
(v) Low physical activity was assessed by the question “How often do you practice any of the following activities (dancing, walking, farmer work, or gardening)?” (Duarte et al., 2014). Answers ranged from one to four, respectively, never/almost never, up to three times a month, once a week, and more than once a week. Answers were then recoded as 0 if answers were “once a week” or “more than once a week,” meaning they were active, and 1 for answers “never/almost never” or “up to three times a month,” which were considered not active.

### Statistical Analysis

The descriptive analysis summarized sample characteristics considering sociodemographic aspects, the components of frailty, and the classification of frailty according to Fried’s phenotype (Fried et al., 2001). Results were displayed using absolute and relative frequencies or central location and dispersion measures, according to the type of variable. To detect and explore relationships between the five components of frailty (active variables), age, sex, and education (supplementary variables), a MCA was performed using R software and the packages FactoMineR and factoextra. Supplementary variables are not used for the determination of the principal dimensions. Their coordinates are predicted using only the information provided by the performed MCA on active variables, i.e., the five components of frailty (Lê et al., 2008).

Multiple correspondence analysis is a multivariate technique designed to discover both interrelations and intra-relations of two or more categorical variables by reviewing the closeness and remoteness between the variables, which allows the analysis of patterns of relationships of several categorical dependent variables. MCA facilitates the interpretation of categorical variables in the cross tables providing information about the similarities, divergences, and associations between the row and column variables. In MCA, some discrimination measures are usually analyzed such as inertia, which measures how far the categories are spread out from the origin, and the eigenvalues, which are the percentage of inertia explained. MCA also allows the graphical representation of the associations in a lower-dimensional space, aiding the interpretation of results. Each variable is represented with a dot in a multi-dimensional space. Dots close to the X or Y axes are highly related with the respective dimension, and those close to each other are considered similar to or related to each other, depending on the areas they fall into. Similarly, dots far from each other are considered to be unrelated (Greenacre, 1988; Anderson, 1994). To define the number of dimensions to retain, the following criteria/considerations were employed: (i) inclusion of MCA dimensions with inertia above 0.2 and (ii) scree test (Hair et al., 1998). In interpreting the discrimination measures and the visual outputs from MCA, the aim should be to identify those components that cluster together.

## Results

Participants (*N* = 142) had a mean age of 88.07 years (*SD* = 5.30 years) and were mainly women (73.9%), and the majority had a low educational level (34.5% were illiterate, and 65.5% had one or more years of school) (Table 1). According to the frailty phenotype (Table 2), 5 (3.5%) individuals were considered robust, 35 (24.7%) were pre-frail, and 102 (71.8%) frail.

**TABLE 1:** Sociodemographic information about participants.

	***N* (%)**
Age years, M (SD)	88.07 (5.30)
**Sex**	
Male	37 (26.1)
Female	105 (73.9)
**Marital status**	
Married/unmarried couples	47 (33.1)
Widow(ed)	86 (60.6)
Single/divorced	9 (6.3)
**Education level**	
Illiterate	49 (34.5)
≥1 year	93 (65.5)

**TABLE 2:** Phenotype of frailty assessment.

	**Total**	**Pre-frail *n* = 35 (24.7%)**	**Frail *n* = 102 (71.8%)**
**Handgrip strength**			
<13.6 kg	93 (65.5)	7 (20.0)	86 (84.3)
≥13.6 kg	49 (34.5)	28 (80.0)	16 (15.7)
**Gait speed**			
≥16.8 s	122 (85.9)	23 (65.7)	99 (97.1)
<16.8 s	20 (14.1)	12 (34.3)	3 (2.9)
**Exhaustion**			
Yes	74 (52.1)	7 (20.0)	67 (65.7)
No	68 (47.9)	28 (80.0)	35 (34.3)
**Physical activity**			
Never/almost never or up to three times a month	113 (79.6)	19 (54.3)	94 (92.2)
Once a week or more than once a week	29 (20.4)	16 (45.7)	8 (7.8)
**Unintentional weight loss**			
≥5%	21 (14.8)	1 (2.9)	20 (19.6)
<5%	121 (85.2)	34 (97.1)	82 (80.4)
**Number of frailty components**			
0	5 (3.5)	−	−
1	13 (9.2)	13 (37.1)	−
2	22 (15.5)	22 (62.9)	−
3	50 (35.2)	−	50 (49.0)
4	44 (31.0)	−	44 (43.2)
5	8 (5.6)	−	8 (7.8)

Considering the phenotype components of the total sample, 93 participants (65.5%) revealed weakness, 122 (85.9%) revealed slowness, 74 (52.1%) reported exhaustion, 113 (79.6%) reported low physical activity, and 21 (14.8%) revealed unintentional weight loss. Specifically, from the pre-frail participants, 13 scored on one component (representing 9.2% of the total of the sample and 37.1% of the pre-frail individuals), and 22 scored on two components (representing 15.5% of the total of the sample and 62.9% of the pre-frail individuals). The most relevant component was gait speed (65.7%), followed by physical activity (54.3%).

Considering the participants labeled as frail, 50 participants scored on three components (35.2% of the total of the sample and 49.0% of the frail individuals), 44 scored on four (31.0% of the total of the sample and 43.2% of the frail individuals), and 8 scored on five components (5.6% of the total of the sample and 7.8% of the frail individuals). Likewise, in participants labeled as pre-frail, the most relevant components were gait speed (97.1%) and physical activity (92.2%).

Our results also showed that of the 62 participants excluded from the analysis, 32 were completely unable to cooperate due to cognitive impairment (e.g., dementia cases, stroke), and 23 due to disability (e.g., stroke consequences, severe hearing impairment) that hampered data collection of some components of frailty. The other seven excluded participants showed tiredness or refusal to perform the some component assessment.

**FIGURE 1:**
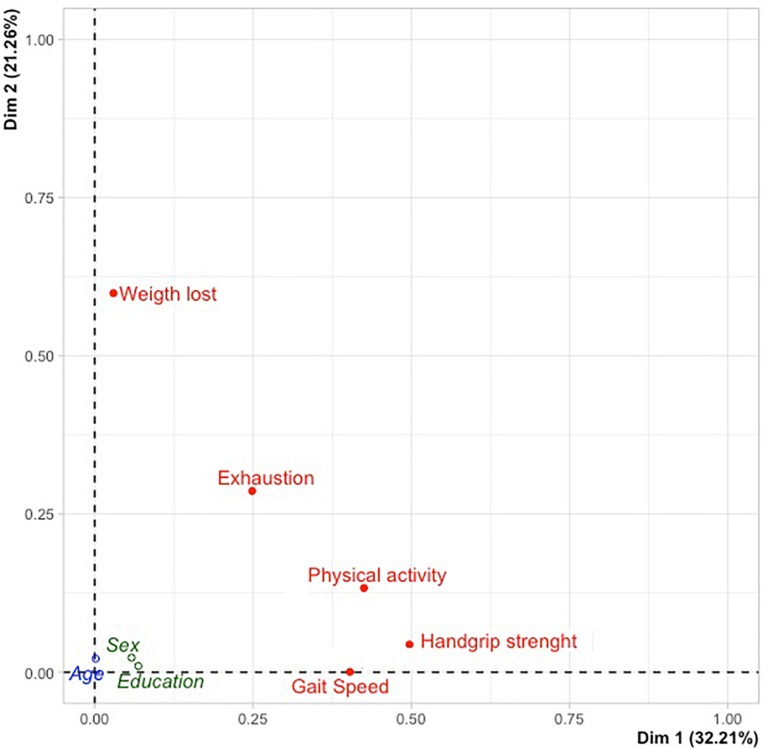
Multiple correspondence analysis (MCA) dimension discrimination measures.

From the MCA analysis, a two-dimension solution was considered the most adequate (Table 3). The first and second dimensions showed, respectively, 0.32 and 0.21 of inertia (Table 3). The first dimension explained 32.21% of the variance, and dimension two explained 21.26% of the variance (Figure 1). Together, both dimensions explained 53.47% of the variance (Table 3). Table 4 describes the MCA dimension discrimination measures. For dimension 1—labeled by us as the functional dimension—the most discriminant variables were weakness, followed by low physical activity and by slowness. Regarding dimension 2—labeled by us as the intrinsic condition dimension—the most discriminant variables were unintentional weight loss and exhaustion (Table 4). Considering the sociodemographic variables tested in MCA (age, sex, and education level), we verified a slight relation of each of them with the two dimensions. Age was almost exclusively related with dimension 2, and sex and education level with dimension 1 (Table 4 and Figure 1).

**TABLE 3:** Inertia and eigenvalues on the dimensions of multiple correspondence analysis (MCA).

	**Dimension 1**	**Dimension 2**	**Dimension 3**	**Dimension 4**	**Dimension 5**
Inertia	0.32	0.21	0.19	0.15	0.13
% Variance	32.21	21.26	18.74	14.53	13.26
Cumulative% variance	32.21	53.47	72.21	86.74	100.00

**TABLE 4:** MCA dimension discrimination measures.

	**MCA dimension 1**	**MCA dimension 2**
Handgrip strength	0.50	0.04
Physical activity	0.43	0.13
Gait speed	0.41	0.00
Weight lost	0.03	0.60
Exhaustion	0.25	0.29
**Supplementary variables**		
Age^a^	–0.04	–0.15
Sex^a^	0.06	0.02
Education level^a^	0.07	0.01
% of variance	32.21	21.26

*^*a*^Supplementary variable.*

## Discussion

In accordance with previous studies (Lewis et al., 2018), our findings revealed a high number of women and widow(ed) participants. The education level among this group is very low (or inexistent), which is why we considered participants who were illiterate versus those who attended school for 1 year or more. This last characteristic is still expressed in the oldest old Portuguese population, as formal education became mandatory only in 1950 for men and in 1960 for women, justifying the high number of participants with low educational level and who were illiterate (Palma et al., 2003).

Concerning the frailty condition, five key aspects emerged from our results. First, we observed a great number of pre-frail and frail subjects. Frail individuals represented more than two-thirds of the total sample (71.8 vs. 24.6% of pre-frail). These results are in accordance with other studies (Duarte and Paúl, 2015; Lewis et al., 2018), which also had a great number of frail oldest old individuals in their samples, highlighting the low differentiation (almost all frail persons) provided by the frailty phenotype of Fried among oldest old individuals and emphasizing the need to better understand its components.

Second, the number of frailty components impaired (Table 2) provided useful information on the “level” of frailty within both the pre-frail and frail groups. Specifically, in the first group, we observed that participants scored mostly in the upper limit of the pre-frail condition (i.e., two components), whereas in the second group, we found that participants scored mostly in the lower and middle limit of frailty (i.e., three and four components, representing a total of 92.2% of frail participants).

Third, the methodological approach using MCA for the study of frailty components proved to add an important understanding for the study of frailty in the oldest old participants. On one hand, it revealed weakness as the most discriminant component for functional dimension (with higher variance explained, Figure 1), evidenced by the fact that among the five components of frailty, weakness was the one with the highest association with the frailty phenotype. On the other hand, MCA identified two main features of frailty: one more related with functionality/physical features (motor behavioral and grip strength) composed of weakness, low physical activity, and slowness; and a second one related with intrinsic conditions (unintentional weight loss and exhaustion). The presence of a functional dimension related with physical features might suggest that these components are potentially more modifiable than the two other components from the intrinsic condition dimension (unintentional weight loss and exhaustion). This distinction of the two frailty dimensions may be a key aspect for customized interventions since it would help to better define pathways as well as to understand the effect of interventions on individual components of frailty as well as in the overall condition. The literature has shown a high number of studies analyzing the effect of interventions on improving the frailty condition (Cesari et al., 2015; De Labra et al., 2015; Apostolo et al., 2018), although few have evaluated the effect of individual and combined interventions in components of frailty phenotype and/or in reversing frailty. A recent study (Liao et al., 2019) testing the effect of two exercise interventions in pre-frail and frail older individuals proved that both interventions were effective for weakness, slowness, and physical activity (functional dimension) but not for exhaustion and weight loss (intrinsic condition dimension), corroborating our results. A previous study by Ng et al. (2015) that conducted a randomized controlled trial among older adults to verify the effects of nutritional, physical, cognitive, and combined interventions on frailty reversal found that the components of frailty benefit from targeted interventions such as physical, nutritional, and cognitive, and especially combined ones. A combined intervention seemed to produce the best effects in almost all components of frailty, except for weight lost, which presented some change in the short and middle term depending on the intervention analyzed but without long-lasting effects. Improvements decreased at 12 months, whatever the intervention performed, which may suggest that this component is effectively an intrinsic aspect and more difficult to change. These results may also have two main implications in the interpretation of frailty: (i) its potential of reversibility (Canevelli et al., 2017), since components from functional condition may have higher reversal rates than intrinsic condition components (probably less changeable or urging other types of intervention, including, namely, nutrition, cognition, and social); and (ii) its relation with practical aspects, namely, in terms of individuals’ assessment (greater attention to components of frailty rather than to the overall score) and in defining and customizing interventions (suitability and adequacy).

Fourth, the slight association of sociodemographic variables with the two dimensions suggested that this approach of frailty showed very little association with sociodemographic aspects not corroborating previous studies (Bieniek et al., 2016; Nyunt et al., 2017; Papachristou et al., 2017; Lewis et al., 2018), which should be the subject of further research, considering these two dimensions of the frailty phenotype and across different age groups. This study analyzed only the oldest old people with very low variability in education level and health condition, as referred to in the Limitations section.

Fifth, more attention should be given to the great number of individuals excluded from the total sample. Participants were excluded due to their total or partial inability to perform the test of components of frailty (due to auditory deficits, consequences of stroke, and dementia, among others). According to Lewis et al. (2018), the frailty phenotype of Fried requires a certain level of functioning, which is in accordance with what we observed in our study once we had to exclude from our analysis a high number of individuals (55 individuals were considered as not having that “certain level” of functioning). In Fried’s original study, that “certain level” of functioning was assured, defining a set of exclusion criteria (e.g., history of Parkinson’s disease, stroke, dementia), missing information about the excluded participants in terms of disability level (total or partial), and the components impaired. Probably, at this advanced age, many of the participants were already dependent (with an irreversible condition and not frail). This should be further explored so that the frailty condition becomes more clear and useful to inform interventions. The distinction between the inability to perform a certain task or requirement and a missing value seems crucial to fully understand the frailty condition.

Overall, the results obtained in this study substantiate the need of a discriminant approach to the frailty phenotype, namely, among very old individuals, bringing into consideration the specific relevance of the different components of frailty (functional dimension and intrinsic condition). The subdivision of the frailty phenotype into two dimensions may help professionals to identify if the frail condition is more related with physical features or with intrinsic aspects, leading to the customization of interventions and bearing in mind that functional aspects are potentially more modifiable than intrinsic ones.

### Limitations

Some limitations must be mentioned. First, our study might benefit from another reference process for participants. The identification of the target population through NGOs could contribute to higher participants disability levels. Second, this study included very old individuals (mean age of 88 years), who could have a higher incidence of health-related problems. We therefore suggest further studies among other younger age groups to test MCA and to verify if the two-dimension approach to frailty remains useful. Further research should also consider studying frailty in those who cannot be fully assessed by means of Fried’s frailty phenotype. In particular, some studies (Ravindrarajah et al., 2013; Payne et al., 2017) demonstrated that those participants who cannot complete the Fried phenotype requirements should be considered frail or dependent (irreversible condition) and had a higher mortality rate than those who could be assessed. Despite these limitations, our results may represent an improvement to the study and conceptualization of the frailty phenotype as well as to the planning of interventions for pre-frail and frail individuals.

## Data Availability Statement

The study was approved by the Ethical Committee of the Institute of Biomedical Sciences of Abel Salazar, University of Porto (process No. 188/2017) and authorized by the Portuguese Data Protection Authority (approval No. 1338/2017), guaranteeing anonymity, privacy, and confidentiality.

## Ethics Statement

The studies involving human participants were reviewed and the study was approved by the Ethical Committee of the Institute of Biomedical Sciences of Abel Salazar, University of Porto (process No. 188/2017) and authorized by the Portuguese Data Protection 396 Authority (approval No. 1338/2017). The patients/participants provided their written informed consent to participate in this study.

## Author Contributions

SA was responsible for the study design, collecting, analyzing, and interpreting data, and manuscript drafting and revision. LT managed, analyzed, and interpreted the data. OR was responsible for study supervision and manuscript revision. CP was responsible for study supervision and made manuscript revisions.

## Conflict of Interest

The authors declare that the research was conducted in the absence of any commercial or financial relationships that could be construed as a potential conflict of interest.
